# Anisotropic hydrogen diffusion in α-Zr and Zircaloy predicted by accelerated kinetic Monte Carlo simulations

**DOI:** 10.1038/srep41033

**Published:** 2017-01-20

**Authors:** Yongfeng Zhang, Chao Jiang, Xianming Bai

**Affiliations:** 1 Fuels Modeling and Simulations, Idaho National Laboratory, Idaho Falls, ID, 83415, USA; 2 Materials Science and Engineering, Virginia Tech, Blacksburg, VA, 24061, USA.

## Abstract

This report presents an accelerated kinetic Monte Carlo (KMC) method to compute the diffusivity of hydrogen in hcp metals and alloys, considering both thermally activated hopping and quantum tunneling. The acceleration is achieved by replacing regular KMC jumps in trapping energy basins formed by neighboring tetrahedral interstitial sites, with analytical solutions for basin exiting time and probability. Parameterized by density functional theory (DFT) calculations, the accelerated KMC method is shown to be capable of efficiently calculating hydrogen diffusivity in α-Zr and Zircaloy, without altering the kinetics of long-range diffusion. Above room temperature, hydrogen diffusion in α-Zr and Zircaloy is dominated by thermal hopping, with negligible contribution from quantum tunneling. The diffusivity predicted by this DFT + KMC approach agrees well with that from previous independent experiments and theories, without using any data fitting. The diffusivity along <c> is found to be slightly higher than that along <a>, with the anisotropy saturated at about 1.20 at high temperatures, resolving contradictory results in previous experiments. Demonstrated using hydrogen diffusion in α-Zr, the same method can be extended for on-lattice diffusion in hcp metals, or systems with similar trapping basins.

The diffusion of hydrogen in hcp metals has attracted extensive research interests for both its scientific merit and technological importance. It is the rate-limiting step for solid hydrogen storage in Mg based alloys^1^ and for mechanical property degradation in Ti^2^ and Zr alloys^3^. For instance, Zr-based alloys such as Zircaloy2 and Zircaloy4 (denoted as Zircaloy) are widely used as the cladding of fuel rods in nuclear reactors for their excellent corrosion resistance and very low absorption cross-section of thermal neutrons. During their service time, these alloys operate in extremely harsh environments, combining high temperatures and corrosive coolants such as water. In light water reactors, Zircaloy claddings directly contact coolant water, with a local temperature at the interface of about 400 °C. Consequently, Zircaloy react with water, producing an oxide layer at the interface and hydrogen (H), of which a substantial fraction infiltrates into the cladding interior^3^. Due to the low solubility of H in α-Zr, which is the main component of claddings, hydrides precipitate in the cladding matrix^4^,^5^,^6^. Hydride formation can detrimentally affect the cladding integrity primarily in two ways. First, the formation of hydrides reduces the fracture toughness of the cladding matrix^7^,^8^, increasing the probability of fuel failure when fuel-cladding mechanical interaction (PCMI) occurs during fuel operation. Second, upon cyclic thermal and mechanical loadings, hydrides can dissolve and reorient. During used-fuel storage, fracture may propagate via the so-called delayed-hydride-cracking (DHC) mechanism, which is induced by the dissolving of matrix hydrides and their subsequent re-formation at crack tips^3^,^9^. To mitigate these detrimental effects requires fundamental understanding of the thermodynamics and kinetics governing hydrogen uptake and hydride formation. In this work, we focus on H diffusion, which is the rate-limiting factor for both hydrogen uptake and DHC.

In the past, understanding of hydrogen diffusion in α-Zr and Zircaloy has relied mostly on experimental measurements^10^,^11^,^12^,^13^,^14^,^15^. It has been commonly accepted that H diffuses in Zircaloy via the same mechanism as in α-Zr, with additional trapping caused by alloying elements and impurities. Due to the differences in the measuring methods and the preparation of samples, scattered data for H diffusivity^10^,^11^,^12^,^13^,^14^ and contradictory results regarding the diffusion anisotropy have been reported. Due to the hexagonal symmetry of α-Zr, the diffusivity of H along <c> usually differs from that along <a> (in a plane parallel to <a> and normal to <c>), with the former suggested to be higher than the latter by Kearns *et al*.^14^. However, in a more recent experiment, the latter was reported to be over an order higher than the former^15^. In addition to these experiments, atomic scale studies such as density functional theory (DFT) calculations have also been applied to H diffusion in α-Zr^16^,^17^,^18^. At high temperatures, H diffuses in metals via thermally activated hopping with the hopping rates described by the transition-state-theory (TST). At low temperatures, due to the small mass of H, the contribution from quantum tunneling may become significant^16^,^19^. The transition temperature is not clear for H in α-Zr. The contribution of thermal hopping at high temperatures can be estimated provided the hopping rates for all involved hopping paths are known. To fully assess the effect of quantum tunneling requires information on the potential energy surface (PES) of H interstitials at energy minima and transition states (TSs) for each hopping path. An alternative approach is to use the semiclassically corrected harmonic-transition-state-theory (SC-HTST) which assumes that the PES is harmonic and requires only the potential energies and vibration frequencies at energy minima and TSs, instead of the full PES^20^,^21^. The effect of tunneling is accounted for by a correction to the classical thermal hopping rates. For H in α-Zr, the information needed for the SC-HTST approach is not yet available.

H diffusion in α-Zr involves multiple hopping paths coupled with each other for long-range diffusion. The hopping rates obtained from DFT calculations need to be incorporated into either analytical theories or other modeling methods such as kinetic Monte Carlo (KMC) to predict diffusivity. In the literature, several theories have been proposed for H diffusion in hcp crystals^22^,^23^. These theories give different predictions for H diffusivity. Therefore, they need to be testified to resolve the discrepancies. In this work, we use lattice KMC parameterized by DFT calculations to compute H diffusivity in α-Zr. The KMC method is well known to be capable of providing atomic scale insights on kinetic processes in crystalline solids^24^. In particular, for diffusion on a pre-defined lattice with known hopping rates, KMC is capable of predicting the diffusivity in cases involving multiple jumps and random trapping sites^25^,^26^. One challenge to apply KMC to H diffusion in hcp metals is that the PES usually contains an energy basin formed by neighboring tetrahedral sites, which strongly limits the efficiency of KMC at low temperatures due to basin trapping. Since accurate H diffusivity is highly desired at low temperatures to predict the kinetics of DHC, which is active in the temperature range of about 150 to 300 °C^27^, acceleration of KMC is needed. Although guidance for general acceleration approaches already exists in the literature^28^, analytical solutions that directly apply to interstitial diffusion in hcp crystals are yet to be derived.

The objectives of this paper are threefold: (i) to develop an accelerated KMC method for H diffusion in hcp metals, (ii) to obtain H diffusivity in Zr and Zircaloy in a wide range of temperatures, and (iii) to resolve the controversy in previous experiments on H diffusion anisotropy in α-Zr. A systematic understanding of H diffusion in α-Zr is expected by accomplishing these objectives. Although in this work the method is demonstrated using Zr and Zircaloy, the same method can be directly applied to other hcp metals and alloys such as Mg and Ti, provided that the required information for hopping rates are available.

## Results

The details of DFT calculations and KMC simulations are provided in the Methods Section. In this section the results are presented.

### Hopping rates and impurity trapping from DFT calculations

**Figure 1:**
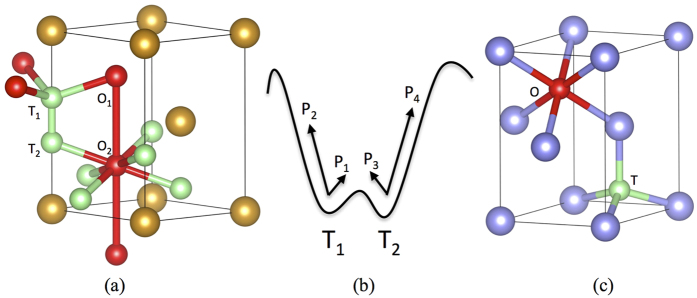
(**a**) The hcp unit cell of Zr (gold) with tetrahedral (green) and octahedral (red) interstitial sites for hydrogen. (**b**) Schematic of an energy basin with two transition states (T_1_ and T_2_). p_1_ (p_3_) represents the probability for the system to transition from T_1_ to T_2_ (T_2_ to T_1_) in next event. p_2_ (p_4_) is the probability for the system to exit the basin from the *left (right*) side in next event. By definition p_1_ + p_2_ = p_3_ + p_4_ = 1. (**c**) 1NN interaction between possible impurity trapping sites (blue) with hydrogen interstitials at tetrahedral and octahedral sites.

For α-Zr, the lattice constants are calculated as *c* = 5.18 Å and *a* = 3.24 Å, agreeing well with previous DFT calculations^17^,^18^,^29^,^30^ and experiments^31^,^32^. Due to its small size, H stays in hcp metals as an interstitial occupying either the tetrahedral (T) site or the octahedral (O) site, as shown in Fig. 1(a). At finite temperatures, both sites are occupied with the partition at thermal equilibrium dictated by the solution energies, and they both are involved in long-range diffusion. Four hopping paths, namely first nearest neighbor (1NN) TT, TO, OT and OO jumps, are found responsible for the three-dimensional diffusion of H. The activation barriers and vibrational frequencies needed to calculate the hopping rates are predicted by DFT calculations, as listed in Table 1. The activation barriers calculated here agree well with those computed by Domain *et al*.^17^ and Christensen *et al*.^18^. In addition to these 1NN jumps, second nearest neighbor (2NN) TT and OO jumps (denoted as TT2 and OO2, respectively) were also suggested in Domain *et al*.^17^. These 2NN jumps are however found to be unstable in this work. Our calculations show that TT2 and OO2 paths spontaneously relax into TO + OT and OT + TO, respectively. Therefore, in our KMC simulations, only 1NN TT, TO, OT and OO jumps are considered. The TT jump has a much lower barrier than others, forming a trapping energy basin in the PES (Fig. 1(b)), which significantly reduces the efficiency of KMC modeling.

Although the absolute solution energy is not needed in KMC simulations, it is calculated in this work for comparisons with the literature. Using Eq. (21) in the Method Section, H solution energy is given as −0.429 eV and −0.366 eV at the tetrahedral and the octahedral site, respectively. Therefore, the tetrahedral site is more stable than the octahedral site for H, with an energetic preference of −0.063 eV, which compares well with the −0.059 eV reported in Domain *et al*.^17^ and −0.086 eV in Burr *et al*.^29^. For a more general comparison, most previous DFT calculations^17^,^18^,^29^,^30^,^33^ have predicted that H prefers the tetrahedral site over the octahedral site based on the solution energy as defined in Eq. (21), which was reported to be −0.60 eV in Domain *et al*.^17^ and Lumley *et al*.^33^, −0.46 eV in Burr *et al*.^29^, and −0.52 eV in Udagawa *et al*.^30^. Experimentally, a value of −0.66 eV was suggested^32^.

It has been pointed out that while evaluating the relative stability of various H interstitial configurations, the contribution of zero-point-energy (ZPE) in the solution energy should be included due to the small mass of H^18^. At 0 K, the ZPE of a H atom at interstitial site *i* is given by 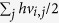. Here *h* is Planck’s constant, and *v*_*i,j*_ the *j*_*th*_ vibration frequency at site *i*. The summation runs over all three vibrational frequencies at energy minima and only the two real frequencies at TSs. Using the results listed in Table 1, the ZPE within the harmonic approximation is found to be 0.134 eV for the octahedral site, and 0.221 eV for the tetrahedral site, respectively. For the ZPE of H at the O site, our result agrees well with the value (0.123 eV) predicted by Christensen *et al*.^18^. With ZPE included, the octahedral site becomes more stable than the tetrahedral site with an energetic preference of 0.026 eV at 0 K. Such a small difference is actually within the uncertainty caused by the harmonic assumption in DFT calculations. It has been shown by Christensen^18^ that by fully taking into account the anharmonic effect, the ZPE at the T site could be reduced from 0.18 eV to 0.166 eV if the neighboring Zr atoms were allowed to relax. A detailed consideration of this anharmonic effect is beyond the scope of this work. Nevertheless, the results indicate that T and O sites are both occupied by H atoms at finite temperatures due to their similar energetic levels.

The binding energies between H and alloying elements such as Sn, Fe, Cr, and Ni are calculated using Eq. (23). The results are listed in Table 2. The concentrations of the alloying elements used in the KMC simulations are also listed. Here, only the 1NN interactions are considered. The 2NN interactions are found to be much weaker than 1NN ones and are therefore neglected. For each type of impurity, three types of 1NN interactions with H exist, depending on the site of H and the relative positions of impurity atoms in reference to the H atom. As shown in Fig. 1(c), H at an octahedral site can be trapped symmetrically by six impurity sites within 1NN distance (number of trapping sites *N*_*t*_ = 6) with a binding energy of 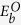. Tetrahedral H interstitials can be trapped by one impurity site along <c> (*N*_*t*_ = 1) with a binding energy of 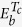, and three others symmetrically (*N*_*t*_ = 3) with 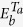. As shown in Table 2, Fe, Cr and Ni are attractive to H. The presence of these alloying elements will increase the overall H solubility and reduce H diffusivity by trapping H atoms locally. Sn is repulsive to H. In particular, H at the tetrahedral sites surrounding a Sn atom is not stable and automatically relaxes into a nearby interstitial site, meaning that these sites are blocked for H diffusion. Accordingly, the rate for H to jump to these sites is set to be zero in KMC simulations. Except for Ni, the trapping energies calculated here are much lower than those given in Christensen *et al*.^18^, which are also listed in Table 2. The comparison is made by comparing with the highest binding energies from our calculations for each type of impurity. As will be shown later by KMC simulations, the large trapping energies given by Christensen *et al*. can lead to much lower H diffusivity in Zircaloy than that reported by experiments.

### Accuracy and efficiency of accelerated KMC

**Figure 2:**
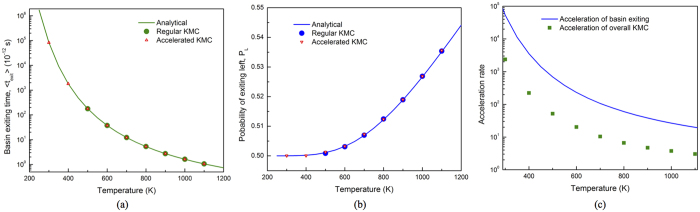
(**a**) Basin exiting time, (**b**) probability of exiting left, and (**c**) acceleration rate as functions of temperature.

The key of our accelerated KMC is to replace the time-consuming T-T jumps spent at the energy basins in KMC with the analytical solutions for basin exiting time and probability, as described in the Method Section. Before presenting the results on H diffusivity, the accuracy and efficiency of the accelerated KMC method are demonstrated. To preserve the diffusion kinetics, it is important to reproduce both the average basin exiting time and the exiting probability for each path. Since the analytical solutions in Eqs (16) and (17) are exact, the acceleration is expected to result in no change in the H diffusion kinetics. To confirm this, KMC simulations are carried out every 100 K from 300 K to 1100 K. In each simulation, 100,000 basin exiting events are used to obtain good statistics. As shown in Fig. 2(a,b), for both exiting time and probability, the results from accelerated KMC perfectly match the analytical solutions and the results from regular KMC as well, indicating the correctness of the analytical solutions and the preservation of diffusion kinetics with acceleration. At 300 K and 400 K, regular KMC is too inefficient to get sufficient sampling of KMC events. Still, the results from accelerated KMC follow the analytical solutions.

It is also interesting to note that at low temperature, the hopping rate ω_1_ ≫ ω_2_ (thus *p*_*1*_ ≫ *p*_*2*_), so that P_L_ ≅ P_R_ as given by Eq. (17). This implies that after a long trapping time in a basin, the memory of the basin-entering site is lost, leading to nearly equal exiting probability to the *left* (from the path H enters basin) and *right* existing path. However, at high temperatures, the trapping time is short and the escaping probability is biased favoring the basin-entering site. Capturing the escaping probabilities is critical for accurate H diffusivity in hcp crystals. Since exiting to the *left* results in zero net displacement along <c>, overestimate (underestimate) of its probability effectively suppresses (accelerates) H diffusion along <c>, leading to error in both overall diffusivity and diffusion anisotropy.

The average basin exiting time depends on temperature, as does the rate of acceleration relative to regular KMC. At low temperatures, *p*_*1*_ approaches unity and the acceleration rate *n*_*acc*_, as defined in Eq. (19) in the Method Section, approaches 1/*p*_*2*_. It should be noted that the acceleration in Eq. (19) applies only to basin exiting. The computing time for jumps outside the basin remains constant. Therefore, the overall acceleration depends on how often the system visits basins and how long it takes to exit a basin by average. For H in α-Zr, the tetrahedral site is frequently visited since its population is twice of that of the octahedral site, with similar energies for both sites. As shown in Fig. 2(c), the acceleration is significant at low temperatures and decreases upon increasing temperature, mainly because of decreasing basin exiting time as given in Eq. (16). At room temperature, over 2,000 times overall acceleration can be obtained. Even at high temperature such as 800 K, the KMC simulations can still be accelerated by a rate of about 10. Since DHC is usually active in the temperature range of about 150 to 300 °C in Zircaloy claddings^27^, acceleration is important for efficient calculations of H diffusivity at low temperatures.

### Hydrogen diffusion in pure Zr

**Figure 3:**
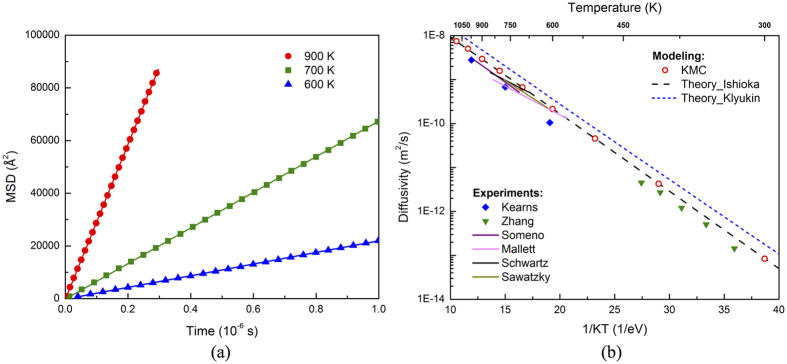
(**a**) Mean-square-displacements (MSDs) of H at 600, 700 and 900 K as functions of time. The symbols are from KMC and lines from linear fitting. (**b**) H diffusivity in α-Zr as a function of inverse temperature, 1/KT.

The three-dimensional H diffusivity in α-Zr calculated by accelerated KMC is plotted in Fig. 3. The calculations are performed from 300 K to 1100 K, one data point every 100 K, covering the temperature range used in previous experiments. To minimize stochastic scattering, at each temperature 100,000 KMC simulations are carried out, each lasting until 100,000 basin exits are detected for sufficient statistics. The averaged mean-square-displacements (MSD) display the expected linear relationship with time (Fig. 3(a)), with the slope proportional to diffusivity. At high temperatures (>600 K), the KMC results are at the upper bound of the scatter in the experimental data, within a factor of 2 different from most experimental results^10^,^11^,^12^,^13^,^14^, as shown in Fig. 3(b). It should be emphasized that the modeling prediction is based on first principles and is completely independent of the experiments (i.e., no data fitting is used). Thus, this work demonstrates the predictive power of computer modeling. The minor discrepancy between KMC and experiments may come from uncertainty in DFT calculations due to underbinding of GGA or that in experiments due to the differences in measuring method and sample preparation. In particular, in Kearns *et al*. polycrystalline samples with various types of impurities were used^14^,^34^. Depending on their interactions with H, impurities and grain boundaries may either facilitate or impede H diffusion. Involving multiple jumps, the diffusivity of H may not necessarily follow the Arrhenius equation. Nevertheless, it is still fitted using the equation *D* = *D*_*0*_exp(−*E*_*m*_*/K*_*B*_*T)* for comparison with experiments, with *D*_*0*_ being the prefactor and *E*_*m*_ the effective migration barrier. The fitting of KMC results gives 5.55 × 10^−7^ m^2^/s and 0.41 eV for *D*_*0*_ and *E*_*m*_, respectively, agreeing well with the experimental values of 6.94–7.82 × 10^−7^ m^2^/s and 0.46–0.47 eV for α-Zr^14^. As will be shown later, better agreement can be achieved by including impurity effects. At low temperatures (<500 K), good agreement between KMC results and experiments^15^ is also observed, with the former being higher but no more than a factor of 2 than the latter. It needs to be pointed out that in ref. 15, H diffusivity was measured separately along <a> and <c>, and the overall diffusivity plotted in Fig. 3(b) is converted using Eq. (13). As will be elaborated later, the results on *D*_*c*_ reported by that work was unreasonably low compared to *D*_*a*_, possibly leading to a low overall diffusivity as well.

Interstitial diffusion in hcp metals involving octahedral and tetrahedral sites has been treated theoretically by different groups. Using an on-lattice random walk model, Ishioka and Koiwa^22^ proposed an algorithm to derive the diffusivities of impurity atoms on a crystal lattice containing multiple sublattices, such as the octahedral and tetrahedral sites in hcp crystals. For H in hcp metals, the diffusivities along <c> and <a> directions are given by:


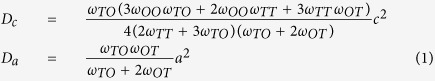


where the hopping rate *ω*_*ij*_ can be calculated using Eq. (5). More recently, starting from Fick’s law and considering the balanced flux between equilibrated tetrahedral and octahedral sites, in Klyukin *et al*. an expression was derived for H diffusivity in hcp metal as^23^:


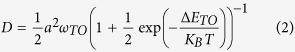


Here Δ*E*_*TO*_ is the difference between the solution energies at the tetrahedral and the octahedral sites, including ZPE. *K*_*B*_ is the Boltzmann constant. Note that in Eq. (2) there is no differentiation of *D*_*c*_ and *D*_*a*_. The parameters listed in Table 1 are used while applying the above theories. As shown in Fig. 3(b), our KMC results agree perfectly with the Ishioka model, which involves all relevant jumps. The Klyukin model is found to overestimate H diffusivity. It ignores the TT and OO jumps and essentially considers only H diffusion along <a>. The overestimation probably comes from the fact that it ignores the time spent for TT and OO jumps.

**Figure 4:**
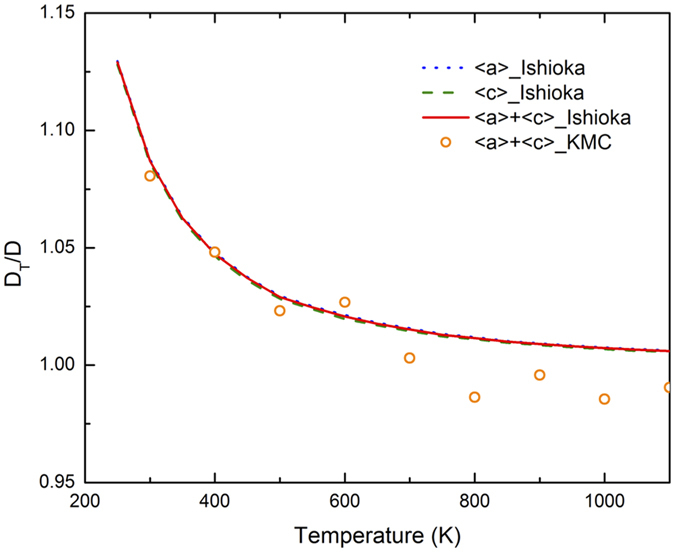
Ratio of tunneling corrected diffusivity (*D*_*T*_) over that without correction (*D*).

The above results are obtained without considering quantum tunneling^19^,^20^, which could be important at low temperatures. Given the relatively high barriers for OO and OT jumps, for H diffusion in α-Zr, tunneling is expected to be less important than in some other metals^16^. According to the SC-HTST theory, the contribution of tunneling becomes significant below a critical temperature, *T*_*c*_ = (*hv*_±_ *E*_*ZP*_/*K*_*B*_)/(2*πE*_*ZP*_ − *hv*_±_ ln2). Here ν_±_ is the imaginary vibrational frequency at the TS (Table 1), and *E*_*ZP*_ the migration barrier with ZPE correction. For TT, TO, OT and OO jumps, the critical temperature is estimated as 72 K, 68 K, 68 K and 38 K, respectively, indicating that tunneling is not significant above room temperature. To better estimate the tunneling effect, Eqs (8) to (10) are applied to the Ishioka theory and in KMC from 300 K to 1100 K. The ratio of tunneling corrected diffusivity (*D*_*T*_) over that without correction (*D*) is plotted in Fig. 4. Since the correction factor in Eq. (9) is always larger than 1, so is the ratio of *D*_*T*_/*D*. Also, *D*_*T*_/*D* decreases with increasing temperature, upon which the tunneling effect diminishes. Above room temperature, the tunneling correction increases H diffusivity by less than 10%, with a similar effect along <c> and <a> and thus negligible change in the anisotropy, as shown in Fig. 4. This indicates that above room temperature, H diffusion in α-Zr is dominated by thermally activated hopping. For this reason, H diffusivity will be calculated without considering the tunneling correction in the rest of the paper.

### Hydrogen diffusion in Zircaloy

**Figure 5:**
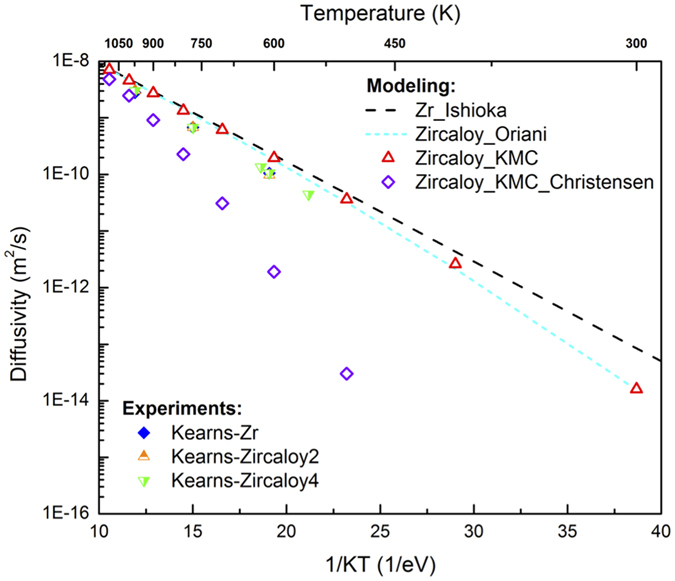
H diffusivity in Zircaloy as a function of inverse temperature, 1/KT.

The diffusivity of H in Zircaloy is plotted in Fig. 5. To better compare with the previous experiments, here the concentrations of Sn, Fe, Cr, and Ni are taken as the average values for Zircaloy2 and Zircaloy4 in Kearns *et al*.^34^, where the exact compositions of the pure-Zr, Zircaloy2 and Zircaloy4 samples were given. The concentrations used in our KMC calculations are listed in Table 2 in unit of atomic percent. As shown in Fig. 5, with all four alloying elements present, the overall hydrogen diffusivity is reduced, with greater reduction at lower temperatures. Fitting of the KMC results using *D* = *D*_*0*_exp(−*E*_*m*_*/K*_*B*_*T*) gives 1.08 × 10^−6^ m^2^/s and 0.46 eV for *D*_*0*_ and *E*_*m*_, agreeing nicely with the averaged values of 7.0 × 10^−7^ m^2^/s and 0.46 eV for α-Zr and Zircaloy from experiments^14^. The higher fitted migration barrier for Zircaloy in reference to that of pure Zr (0.41 eV) is mainly due to the reduction in diffusivity at low temperatures. In the high temperature region (≥600 K), the reduction as shown by the KMC results is less than 15% (mostly within 10%). Such a small reduction is within the experimental scatter, in good agreement with Kearns *et al*. that H diffusivities in Zr, Zircaloy2 and Zircaloy4 are hardly distinguishable from each other^14^. In the previous experiments, the pure Zr samples contained impurities with very dilute concentrations^34^, which may have brought the H diffusion lower and closer to that in Zircaloy. Again, the minor discrepancy between KMC and experiments for Zircaloy could be caused by errors in DFT calculations, or possible presence of other impurities and grain boundaries in experiments.

As shown in Table 2, the binding energies calculated here are quite different from that given in Christensen *et al*.^18^. For a comparison, KMC simulations were also performed with the binding energies taken from ref. 18. As no information about the trapping site was given in ref. 18, in KMC only the trapping of a H atom at a tetrahedral site by a nearby solute atom along <c> direction (with *N*_*t*_ = 1) is considered, so that the least trapping (i.e., minimum reduction of H diffusivity) is expected. Still, significant reduction in H diffusivity is observed using the binding energies in ref. 18 (Fig. 5), in contrast to previous experiments that showed similar H diffusivity in α-Zr and Zircaloy^14^. This suggests that the H-solute binding might be overestimated in Christensen *et al*.^18^, which is possibly due to the neglect of local magnetic moments of Fe and Cr atoms in DFT calculations.

Theoretically, the trapping of impurities can be estimated using the Oriani model^25^,^35^ when the concentrations of the diffuser and impurities are not very high. Specifically, the diffusivity with impurities *D*_*im*_ is given by:


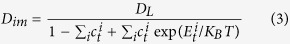


Eq. (3) holds when 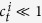. *D*_*L*_ is the diffusivity without impurities. The physical meanings of other symbols are the same as in Eq. (20) in the Method Section. As shown in Fig. 5, the results from KMC simulations agree well with the Oriani model, while applying which the impurity-free diffusivity *D*_*L*_ is obtained using the Ishioka model and the binding energies in Table 2 are used. According to Eq. (3), alloying elements that are attractive to H, such as Fe, Cr and Ni, reduce H diffusivity. In contrast, solutes such as Sn that are repulsive to H slightly increase H diffusivity. The results from KMC on the separate effect of each element are also in good agreement with the Oriani model, which adopts the same assumptions as used in the mean-field KMC method.

### Hydrogen diffusion anisotropy

Due to the hexagonal symmetry of α-Zr, H diffuses anisotropically along <c> and <a> directions, with the latter representing the isotropic 2D diffusion in the basal plane. Since multiple jumps are involved, the anisotropy is not readily evident by just examining the individual hopping rates. KMC simulations allow for the calculation of diffusivity along both <c> and <a> and thus the anisotropy. Moreover, the relative importance of the jump paths can be evaluated at given temperatures to fully elucidate the controversy as reported in previous experiments: *D*_*c*_*/D*_*a*_ > 1 but not exceeding 2 at temperatures above 600 K in Kearns *et al*.^14^, and *D*_*c*_*/D*_*a*_ < 0.1 below 500 K in Zhang *et al*.^15^. Even though these experiments were done in different temperature ranges, the data reported were sufficient to establish contradicting trends over a wide range of temperatures.

**Figure 6:**
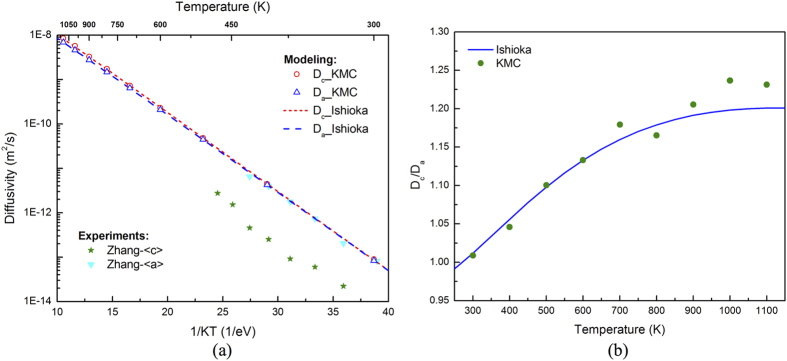
(**a**) H diffusivities along <c> and <a> as functions of inverse temperature, 1/KT. (**b**) *D*_*c*_*/D*_*a*_ ratio as obtained from KMC simulations and from the Ishioka model from 300 K to 1100 K.

In KMC simulations, *D*_*c*_ and *D*_*a*_ can be calculated using Eq. (12) by decomposing the MSD into components along <c> and <a>. The results obtained from KMC simulations follow the Ishioka model well, as shown in Fig. 6(b). Some scatter in the KMC results is observed due to the stochastic nature of the KMC method. The *D*_*c*_*/D*_*a*_ ratio is found to increase with temperature and saturates to about 1.2 at high temperatures (Fig. 6(b)), again agreeing well with the Ishioka model, where the anisotropy is given by:


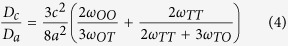


The temperature dependence in *D*_*c*_*/D*_*a*_ comes from the temperature dependent hopping rates. At very low temperatures, *ω*_*OO*_ ≪ *ω*_*OT*_ and *ω*_*TO*_ ≪ *ω*_*TT*_, so that *D*_*c*_*/D*_*a*_ approaches 3*c*^2^/8*a*^2^, which is 1.0 for hcp metals with the ideal *c*/*a* ratio and about 0.96 for α-Zr using the lattice constants from our DFT calculations. This means that at very low temperatures, *D*_*a*_ > *D*_*c*_ for H in α-Zr. Upon increasing temperature, OO jumps become more important and the first term in the parentheses of Eq. (4) increases. Since the migration barrier for TT jump is significantly lower than that for TO jump (see Table 1), the second term in the parenthesis of Eq. (4) remains close to 1 and only slightly decreases with increasing temperature. The overall trend is that D_c_/D_a_ continuously increases with temperature. As a result, at high temperatures *D*_*c*_ becomes higher than *D*_*a*_. The transition between *D*_*c*_/*D*_*a*_ < 1 and *D*_*c*_/*D*_*a*_ > 1 occurs at about 270 K. In the above analysis the anisotropic thermal expansion of α-Zr is ignored since its effect is orders of magnitude lower.

**Figure 7:**
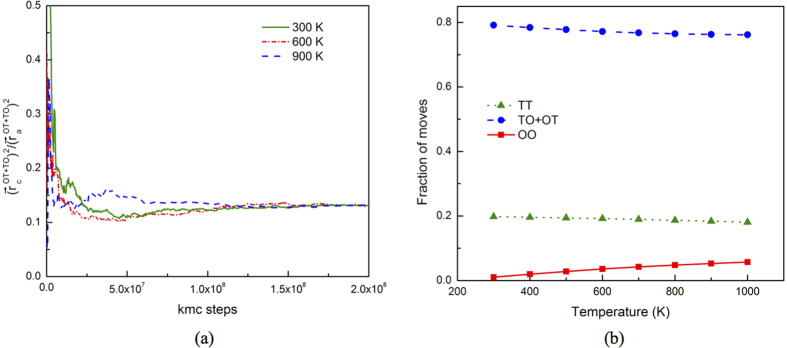
(**a**) Ratio of MSD along <c> over that along <a> induced by TO and OT moves as a function of time at several temperatures. (**b**) Fractions of KMC moves taking TO + OT, TT and OO jumps at various temperatures. Here one TT move represents a basin exit event involving a net TT displacement, not an actual TT move as in regular KMC simulations.

Both KMC and the Ishioka model agree with Kearns *et al*., which predicted *D*_*c*_*/D*_*a*_ > 1 with a ratio no more than 2.0 at temperatures above 600 K^14^. The KMC results on *D*_*a*_ agree perfectly with those measured by Zhang *et al*. (Fig. 6(a)), where pure single-crystal α-Zr samples were used^15^. However, substantial discrepancy on *D*_*c*_ is noticed. In KMC simulations, *D*_*c*_ is not distinguishable from *D*_*a*_ from 300 K to 500 K, while in the experiments the former was reported to be over an order of magnitude lower than the latter^15^ (see Fig. 6(a)). Such a low *D*_*c*_*/D*_*a*_ ratio is very unlikely if the 3-D, on lattice random walk of H is not altered. H diffusion along <a> is via TO and OT jumps, both with a component along <c> and thus contributing to diffusion along <c>. TT and OO jumps contribute only to <c> diffusion but via two different ways: i) the net displacement induced by these two jumps, and ii) providing a path for H to jump from one <a> plane to another, i.e., bridging TO (OT) jumps in neighboring <a> planes, so that H can perform 3-D random walk. Without the bridging effect, H diffusion will be confined in <a> planes, and the <c> component of TO and OT jumps will cancel each other. During 3-D random walk, the geometry of TO and OT jumps sets a lower bound of *D*_*c*_*/D*_*a*_, determined by the ratio of 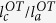; here 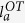 and 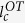 are the components in absolute length of OT jump (or TO) along <a> and <c>, respectively. For H in α-Zr, 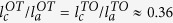, giving a lower bound of 
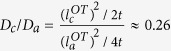
. Such a geometric effect is independent of temperature except for negligible anisotropic thermal expansion. Indeed, as shown in Fig. 7(a), with sufficient KMC jumps, 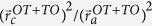 converges to 0.13 regardless of temperature, corresponding to a *D*_*c*_*/D*_*a*_ ratio of 0.26. Here, 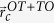 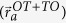 is the total displacement along <c> (<a>) summed over all OT and TO jumps. The KMC analysis also proves our assumption of the 3-D, on-lattice random walk of H, because otherwise 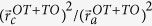 cannot converge to 0.13. Note that this ratio is obtained without including the contribution from TT and OO. Therefore, *D*_*c*_*/D*_*a*_ should be no less than 0.26 as long as H performs 3-D random walk, suggesting that in Zhang *et al*., *D*_*c*_ was likely be measured at a situation where H diffusion along <c> was suppressed, deviating the random walk behavior. Given the small barrier of TT jump, confined diffusion along <a> is very unlikely in α-Zr or Zircaloy in the dilute concentration regime. Actually, our KMC simulations show that regardless of temperature, TT jumps that bridge two neighboring <a> planes accounts for about 20% of all KMC jumps (Fig. 7(b)), and provides an effective path bridging OT and TO jumps in neighboring <a> planes. Here, each TT jump represents a basin exit event involving a net TT displacement, not an actual TT move as in regular KMC. With increasing temperature, OO jump becomes more important in contributing to <c> diffusion, for its increasing fraction of jumps and long absolute length.

The unreasonably low *D*_*c*_*/D*_*a*_ ratio measured in Zhang *et al*.^15^ may be explained by the so-called blocking layer effect observed in experiments on H in hcp Mg^36^. In Zhang *et al*., H diffusivity was measured using the surface segregation approach^15^. It is possible that although the matrix H concentration was kept below the solubility limit, the local H concentration near surface could have exceeded that limit when surface segregation occurred. Consequently, precipitation of hydrides such as coherent hydride clusters^37^ may have occurred. As the hydrides habit on basal planes with small thickness along <c>, they block H diffusion along <c>, reducing *D*_*c*_ without affecting *D*_*a*_ much, similar to the situation for H in hcp Mg^36^.

## Discussion

In this section, some discussion is given regarding the comparison between the present results with previous experiments and theories, as well as the applicability of the accelerated KMC method in other material systems.

The diffusivity of H in α-Zr predicted by KMC is at the upper bound of the scatter in the experimental data. The minor discrepancy, which is within the experimental error limit, may come from uncertainties in DFT calculations or in previous experiments. While calculating H vibrational frequencies using DFT, it is assumed that the vibration of H can be decoupled from that of the α-Zr lattice due to the large difference in their masses. Specifically, Zr atoms are fixed in these calculations since their vibration frequencies are orders lower. Minor error might be induced by such a treatment. For instance, an error of about 0.30 THz (~10 cm^−1^) was identified for the vibrational frequencies of H (at the order of ~30 THz) on a Ni (111) surface by ignoring lattice atom vibration^38^. Similar anharmonic effect has been noticed for H in α-Zr by Christensen *et al*.^18^. We also note that in the diffusivity calculations (Figs 3 and 5), the effect of quantum tunneling is neglected because its contribution is negligible above room temperature. It should be pointed out that the previous experiments are not exempted from uncertainties either due to the differences in measurement method and sample preparation, as indicated by the scatter in results.

For Zircaloy, again the KMC results are higher but within a factor of 2 than those from experiments. Compared to that in α-Zr, the addition of alloying elements slightly reduces H diffusivity, with negligible effect at high temperatures. This is in line with previous experiments, which found similar H diffusivity in α-Zr, Zircaloy2, and Zircaloy4^14^. The mean-field KMC approach for impurity trapping has two major assumptions. First, Eq. (20) is used assuming that the impurity atom located in the proximity of an H atom modifies only the energy of the initial state without altering the TS. This is inaccurate for H hopping around impurity atoms. However, it still captures the effective migration barrier for an H atom to move away from an impurity atom. To confirm this, the diffusion barrier for an H atom to diffuse away from a Ni atom, from a 1NN tetrahedral site to a 2NN tetrahedral site along <c>, is calculated using DFT. The directly calculated barrier is 0.350 eV, very close to that estimated using Eq. (20), 0.341 eV (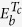, 0.212 eV, plus *E*_m_ for T-T jump, 0.129 eV). Second, in this work only interaction between H and impurity atoms is considered, with no interactions between H atoms and between impurity atoms. Such an assumption applies when the concentrations of H and impurities are low (i.e., dilute solution). For high H concentration or high impurity concentrations, the interactions between H atoms and between impurity atoms need to be included. The mean-field approximation in Eq. (20) needs to be replaced by more accurate calculations of rate parameters considering local atomic configurations. The same assumptions are shared by the Oriani model^35^, which essentially predicts the same results as those from the mean-field KMC.

By comparing KMC with previous theories, this work demonstrates that for hcp metals the Ishioka model is accurate for H diffusivity, and a correction using the Oriani model is sufficient for dilute alloys, provided that all hopping rates are available. However, KMC simulations will be more useful in cases involving spatial heterogeneities, e.g., stress fields induced by cracks and hydrides. The presence of stress fields alters the local solubility and hopping rates as well at each atomic site, making the mean-field approach not applicable.

While the current work focuses on α-Zr and Zircaloy, the same method can be directly applied for H diffusion in other hcp metals such as Mg and Ti and their alloys, where similar diffusion mechanisms and energy basin hold. In Mg, the barrier for T-T jump is about 0.1 eV, much lower than that for T-O (~0.23 eV) and O-O (~0.21 eV) jumps^23^. In Ti, the barrier of T-T jump (0.061 eV) is nearly an order lower than that of T-O (0.424 eV) and O-O (0.625 eV) jumps^2^. H diffusion is critical for H storage in Mg and its alloys^1^, and for hydride embrittlement in Ti-based alloys^2^. In a more general sense, the acceleration method applies to interstitial diffusion in hcp metals, or to problems with similar energy basins^39^ as shown in Fig. 1(b).

## Conclusion

In this work, an accelerated KMC method is developed for efficient calculations of H diffusivity in hcp metals, and demonstrated using H diffusion in α-Zr. Using the hopping rates predicted by DFT, the method accurately predicts H diffusivity in α-Zr and Zircaloy, providing reliable data that could be used for upper scale modeling^5^,^6^. The results from KMC agree very nicely with previous independent experiments at various temperature ranges^10^,^11^,^12^,^13^,^14^,^15^ and the Ishioka theory^22^. The perfect agreement between KMC and Ishioka theory validates the correctness of the analytical equations in this theory. The microscopic diffusion mechanisms obtained from DFT and KMC provide a systematic understanding of H diffusion in α-Zr, which helps to resolve the controversy in previous experiments regarding H diffusion anisotropy. Above room temperature, H diffuses by thermal hopping involving 1NN OT, TT, TO and OO jumps. At low temperatures, an effective diffusion path is via OT- > TT- > TO moves, with the contribution of OO increasing with increasing temperature. The diffusion of H is anisotropic, with the *D*_*c*_/*D*_*a*_ ratio increasing from below 1.0 at very low temperatures to about 1.20 at high temperatures. In addition to H diffusion in hcp metals, the accelerated KMC method may be applied in other material systems with similar energy basins.

## Methods

### Residence time lattice kinetic Monte Carlo

In this work we use lattice KMC to calculate the diffusivity of H in Zr. Due to its small atomic size, H stays in Zr as an interstitial, residing in either a tetrahedral or an octahedral site, as shown in Fig. 1(a). A H atom at a tetrahedral site, T_1_ for instance, can jump to the neighboring T_2_ site (TT jump) or one of the three neighboring O sites (TO). A H atom at an octahedral site, say O_2_, can jump to one of the two neighboring O sites (OO), or one of the six neighboring T sites (OT). All these jumps are involved in three-dimensional diffusion. According to the quantum corrected harmonic transition state theory^40^, the hopping rate from site *i* to *j* is given by:


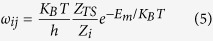


Here *T* is temperature; and *h* is the Planck constant. *E*_*m*_ is the classical migration barrier associated with the *i*- > *j* path. *Z*_*TS*_ and *Z*_*i*_ are the partition functions for the transition state (TS) and the initial state. Their ratio accounts for the zero-point energy correction and it is given by:


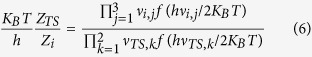


where *f(x*) = sinh (*x*)/*x*. Note that Eq. (6) takes into account the ZPE correction at low temperatures and also reproduces the classical hopping rate at high temperatures.

In Eq. (6) *v*_*i,j*_ (*v*_*TS,k*_) is the *j*^*th*^ (*k*^*th*^) vibrational frequency of H at site *i* (TS). For each jump, the migration barriers and vibrating frequencies are calculated using DFT calculations at 0 K. The results are listed in Table 1. At each KMC step, the hopping rates are calculated using Eq. (5) for all possible moves. A random number is drawn to pick one jump from the list. The time is advanced following the residence time algorithm by:


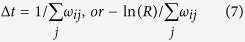


with *R* being another random number between 0 and 1. The use of a random number in Eq. (7) better mimics the stochastic nature of kinetic events. With sufficient sampling, the two expressions in Eq. (7) converge to each other.

The above formulation describes diffusion via thermally activated hopping without considering quantum mechanical tunneling, which has been shown to have substantial contribution to H diffusion in some metals at low temperatures^16^. Quantitative prediction of tunneling requires time-consuming calculations of the PES of H interstitial for each jump. Alternatively, assuming that the PES is harmonic at TSs and energy minima, the effect of tunneling can be estimated by applying a semiclassical correction to the hopping rates following the SC-HTST^20^,^21^, given by:


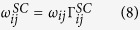


Here 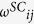 is the corrected hopping rate with quantum tunneling, accounted for by the coefficient 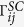. At a given temperature T, 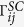 is given by:


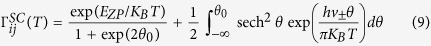


In Eq. (9), *E*_*ZP*_ is the ZPE corrected migration barrier, by


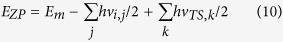


and *v*_*±*_ is the imaginary vibration frequency at the transition state, given as negative in Table 1. The upper limit of the integration is given by *θ*_0_ = *πE*_*ZP*_/*hv*_±_.

To obtain diffusivity, the mean-square-displacement (MSD) of the migrating H atoms at a time *t* is calculated by:


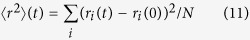


where *N* is the number of total H atoms in a KMC simulation, and *r*_*i*_(*t*) and *r*_*i*_(0) are the atomic positions of the *i*^*th*^ H atom at time *t* and time *0*, respectively. When multiple H atoms are used in a simulation, they are treated as non-interacting with each other and allowed to overlap, to give the diffusivity in the dilute concentration regime.

Decomposing the total MSD into that along <c> and <a>, the diffusivities along <c>, *D*_*c*_, and <a>, *D*_*a*_, can be calculated by:


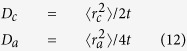


The different coefficients in the denominator for *D*_*c*_ and *D*_*a*_ are due to the fact that *D*_*a*_ is two-dimensional in <a> plane (normal to <c>), while *D*_*c*_ is one-dimensional along <c>.

The three-dimensional diffusivity is given by:


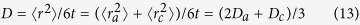


By plotting the MSD as a function of time, diffusivities at various temperatures can be obtained by linearly fitting the MSD curves. Usually, averaging over a large number of independent KMC runs is needed to minimize the stochastic effect.

### Acceleration of KMC

For H in hcp metals, the migration barrier of TT jump is usually much smaller than that of TO, OT and OO, forming an energy basin (Fig. 1(b)). At low temperatures, in regular KMC simulations a large fraction of the KMC moves are the back and forth moves between 1NN tetrahedral sites, with zero contribution to the long-range diffusion. This drastically affects the simulation efficiency, assuming that each KMC step costs about the same CPU time. (This assumption holds well for the case here. In general it depends on the number of events at each KMC step and the complexity to calculate the rates of all events). In general, the energy basin problem can be overcome by solving the master equations of the absorbing Markov chain for the occupation probability of the system as a function of time for each transition state^28^. The solutions can be used to replace regular KMC events to improve the efficiency. Following the same method, the expected exiting time and exiting probability for energy basin containing two energy minima (Fig. 1(b)) are derived as:


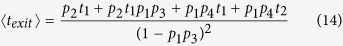


and


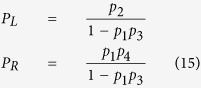


Here *P*_*L*_ is the probability for the system to exit to the *left*, i.e., the site from where it enters the basin, and *P*_*R*_ that to the *right*. By definition *P*_*L*_ + *P*_*R*_ = 1 once the system exits the basin. *t*_*1*_(*t*_*2*_) are the residence time at the T_1_ (T_2_) states before next event occurs, which is the time step in a KMC simulation as given in Eq. (7). *p*_*1*_ (*p*_*3*_) represents the probability for the system to transition from T_1_ to T_2_ (T_2_ to T_1_) in next event. And *p*_*2*_ (*p*_*4*_) is the probability for the system to exit the basin from the *left (right*) side in next event. By definition *p*_*1*_ + *p*_*2*_ = 1 and *p*_*3*_ + *p*_*4*_ = 1.

In pure hcp crystals, the two neighboring T sites (T_1_ and T_2_ in Fig. 1(b)) are symmetrical and so are the O sites (for each exit in Fig. 1(b) there are three symmetrical O sites) for the H atom to exit to. Therefore, we have *p*_*1*_ = *p*_*3*_ and *p*_*2*_ = *p*_*4*_. Similarly, *t*_*1*_ = *t*_*2*_ = *t*_*0*_. Eqs (14) and (15) can thus be reduced to:


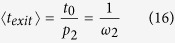


and


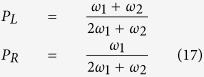


with *ω*_*i*_ being the hopping rate of the *i*th jump, as given by Eq. (5). As can be seen from Eq. (17), a H atom always exits with a higher probability from the tetrahedral site where it enters the basin, since *P*_*L*_ > *P*_*R*_ always holds. In cases of *ω*_1_ ≫ *ω*_2_, e.g., at low temperatures, *P*_*R*_ approaches *P*_*L*_, giving nearly equal probabilities to exit from both T sites. To assure that the diffusion kinetics is preserved during acceleration, it is critical to capture the probability of each exiting path in addition to the basin exiting time. It is also interesting to note from Eq. (16) that for this case the expected exiting time is independent of the barrier between the two minima in the basin.

For acceleration, the KMC events in basins are replaced using the solutions in Eqs (16) and (17). In a KMC simulation with only one H atom, once it enters a basin, it will be taken out in next event, with the exiting path chosen according to Eq. (17). The time advancement is:


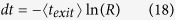


where *R* is another random number. Statistically, a H atom in a basin will perform 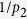 moves before exiting. Assuming that each KMC step consumes the same CPU time, the acceleration rate of basing exiting time can estimated by the below equation:


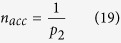


The above approach is for KMC simulations with one H atom in each. In simulations with multiple H atoms, the same acceleration can be achieved by combining neighboring tetrahedral sites (T_1_ and T_2_ in Fig. 1(b) for example) into a *super* site (T^*^), and modifying the hopping rates towards neighboring octahedral sites based on the path along which a H atom enters the combined T^*^ site. We note that since the time step is inversely proportional to the number of H atoms used in the simulation, the use of multiple non-interacting H atoms results in no change in the diffusion kinetics and theoretically no change in the efficiency either.

### Mean-field impurity trapping

To consider impurity trapping, the mean-field KMC approach^25^,^26^ developed for cubic lattice is extended to hcp, where each impurity atom can trap H in three different ways with different binding energies (see Fig. 1(c)). At each KMC step, the migration barrier of each jump is modified by:


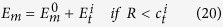


Here 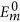 is the barrier without trapping. 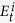 is the binding energy of H at a trapping site *t* with a concentration 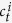, induced by impurity *i*, whose concentration is *c*_*i*_. *R* is a random number to be drawn each time when the jump rate is evaluated. The concentration of trapping site *t* is given by 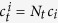, with *N*_*t*_ being the number of trapping sites induced by an impurity atom.

### DFT calculations

*Ab initio* calculations are performed using the all-electron projector augmented wave method within the generalized gradient approximation of Perdew, Burke, and Ernzerhof (PBE-GGA)^41^, as implemented in VASP^42^. Large 96-atom supercells, which can be constructed from a 4 × 4 × 3 extension of the 2-atom hcp Zr unit cell, are used throughout our calculations in order to minimize the unphysical interactions between a H atom and its periodic images. A high plane-wave cut-off energy of 500 eV and a dense 5 × 5 × 5 Monkhorst–Pack *k*-point mesh are used to ensure high numerical accuracy for total energy calculations. All internal atomic positions are fully optimized using a conjugate gradient method until forces are less than 0.01 eV/Å. Further relaxations of supercell volumes have been found to have negligible effect on the final results.

Following Domain *et al*.^17^, we calculate the H solution energy in Zr as:


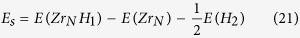


Here *E(Zr*_*N*_*H*_1_) and *E(Zr*_*N*_) are the total energies of a hcp-Zr simulation cell containing *N* Zr atoms, with and without a H atom, respectively. *E(H*_2_) is calculated by placing a single hydrogen molecule in a large 10 Å × 10 Å × 10 Å simulation cell. The equilibrium H-H distance d_H-H_, vibrational frequency v_H-H_, and dissociation energy E_H-H_ of of H_2_ molecule are calculated to be 0.75 Å, 130.1 THz and 4.54 eV, respectively. These values are in excellent agreement with the experimental values^19^ (d_H-H_ = 0.74 Å, v_H-H_ = 130.8 THz, and E_H-H_ = 4.48 eV).

The definition used in Eq. (21) is the classical solution energy without considering the ZPE, which should be included when free energy is of interest. The ZPE corrected solution energy can be estimated as:


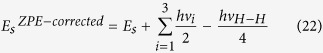


The TSs for all diffusion paths of H are obtained using the climbing image nudged elastic band (CI-NEB) technique^43^ with 3 intermediate images. Here we only consider 1NN TT, TO, OT and OO jumps since the 2NN jumps are found to be unstable and they spontaneously relax into 1NN jumps. The normal-mode vibrational frequencies of H are obtained from the eigenvalues of the Hessian matrix constructed using finite differences with a small displacement of 0.05 Å. All metal atoms are rigidly constrained during such calculations.

The binding energy between a substitutional solute element X (X = Sn, Fe, Cr, Ni) and an interstitial H atom in hcp Zr can be calculated using the following equation:


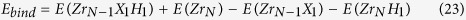


where *E(Zr*_*N*−1_*X*_1_*H*_1_) is the total energy of the supercell containing one impurity atom X and one H interstitial in close proximity of each other. *E(Zr*_*N*_) and *E(Zr*_*N*−1_*X*_1_) are the total energy of the perfect Zr supercell with *N Zr* and the Zr supercell with a substitutional X atom, respectively. *E(Zr*_*N*_*H*_1_) is the energy of a Zr supercell with one H occupying the same type of interstitial site as that in the supercell used for *E(Zr*_*N*−1_*X*_1_*H*_1_). As shown in Fig. 1(c), there exist three different 1NN interactions between H and an impurity within 1NN distance. All these interactions are considered in our calculations. For binding between H and Fe, Cr and Ni, spin-polarized calculations have been performed. According to our calculations, Fe and Cr develop large local magnetic moments (>2 μ_B_), while Ni is essentially non-magnetic. The final results are summarized in Table 2.

## Additional Information

**How to cite this article:** Zhang, Y. *et al*. Anisotropic hydrogen diffusion in α-Zr and Zircaloy predicted by accelerated kinetic Monte Carlo simulations. *Sci. Rep.* **7**, 41033; doi: 10.1038/srep41033 (2017).

**Publisher's note:** Springer Nature remains neutral with regard to jurisdictional claims in published maps and institutional affiliations.

## Figures and Tables

**Table 1:** Activation barriers and vibrational frequencies for 1NN jumps from DFT calculations.

Path	*E*_*m*_ (eV)	ν_i,1_ (THz)	ν_i,2_ (THz)	ν_i,3_ (THz)	ν_ts,1_ (THz)	ν_ts,1_ (THz)	ν_±_ (THz)
TT	0.129 (0.12^17^, 0.129^18^)	36.87	36.87	33.06	43.06	43.04	−17.92
TO	0.406 (0.41^17^, 0.412^18^)	36.87	36.87	33.06	45.80	42.66	−17.59
OT	0.346 (0.35^17^)	23.32	20.84	20.84	45.80	42.66	−17.59
OO	0.398 (0.41^17^, 0.427^18^)	23.32	20.84	20.84	47.30	47.29	−9.95

The activation barriers from refs 17 and 18 are listed in parentheses.

**Table 2:** Concentrations (atomic fraction) of Zn, Fe, Cr, and Ni in Zircaloy and their binding energies (eV) with hydrogen.

Element	Concentration, *c*_*i*_	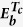	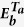	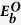
Sn	0.0111	Not stable	Not stable	−0.037 (−0.031^18^)
Fe	0.00268	0.094 (0.58^18^)	0.069	0.106
Cr	0.00173	0.089 (0.269^18^)	0.065	0.085
Ni	0.0003622	0.212 (0.187^18^)	0.153	0.165

Positive (negative) binding energy means attractive (repulsive) interaction.
